# Effect of a Family-Centered Empowerment Model–Based Intervention on the Caregiving Capacity and Preparedness of Caregivers of Children With Malignant Neoplasms: Protocol for a Quasi-Experimental Study

**DOI:** 10.2196/73304

**Published:** 2025-07-29

**Authors:** Xiaowan Li, Yanhua Yang, Qiurong Chen, JingJing Ma, Feng Lu, Xiaoli Luo

**Affiliations:** 1 Department of Pediatric Hematology and Oncology Nursing West China Second University Hospital Sichuan University Chengdu China; 2 Key Laboratory of Birth Defects and Related Diseases of Women and Children (Sichuan University) Ministry of Education Chengdu China; 3 Global Health Nursing Graduate School of Biomedical and Health Sciences Hiroshima University Hiroshima Japan

**Keywords:** caregiving preparedness, family-centered empowerment model, malignant neoplasms, childhood cancer, educational program, depression, anxiety, stress

## Abstract

**Background:**

Malignant neoplasms are among the most common causes of disease-related death in children. Long-term chemotherapy often requires a high degree of parental involvement. Family caregivers’ preparedness and capacity are critical in reducing the burden of care and improving quality of life. This study looks to examine the impact of a family-centered empowerment model (FCEM)–based intervention on the caregiving capacity and preparedness of family caregivers.

**Objective:**

This study aims to develop and evaluate an FCEM-based intervention to improve caregiving preparedness and capacity among family caregivers of children with malignant neoplasms. It also examines the potential effects of the intervention on self-efficacy and psychological outcomes, including depression, anxiety, and stress.

**Methods:**

This quasi-experimental study focuses on caregivers of children with malignant neoplasms attending our hospital for the first time, implementing a 4-phase FCEM-based intervention program evaluated through questionnaires administered 3 days after admission and 3 days before discharge. Differences in caregiving preparedness, caregiving capacity, self-efficacy, and depression, anxiety, and stress scores will be assessed using independent and paired *t* tests, Mann-Whitney tests, and paired rank-sum tests for both within-group and between-group comparisons preintervention and postintervention.

**Results:**

Recruitment will be conducted in 2 waves (control group: July to December 2025; intervention group: July to December 2026). It is expected that the intervention group will show significantly greater improvements in caregiving preparedness, caregiving capacity, and psychological well-being compared to the control group.

**Conclusions:**

The findings of this study are expected to provide evidence for the development of structured family empowerment in pediatric oncology. In the future, expanding to multiple centers and conducting targeted surveys among caregivers of children with different cancer types would help validate and promote the effectiveness of family empowerment interventions.

**Trial Registration:**

ClinicalTrials.gov NCT06810388; https://clinicaltrials.gov/study/NCT06810388

**International Registered Report Identifier (IRRID):**

PRR1-10.2196/73304

## Introduction

### Background

Malignant neoplasms in childhood usually refer to malignant tumors with onset between the ages of 0 and 14 years and are the most common cause of death from childhood illnesses, including leukemia, lymphomas, tumors of the brain and central nervous system, and osteosarcomas [1-3]. Approximately 13.7 million children younger than 15 years are predicted to suffer from tumors by 2050 [4]. In China, approximately 22,000 cases of malignant neoplasms occur in children younger than 15 years each year, and the prevalence rate is estimated to increase annually by 5% [4].

Family caregivers, often parents or grandparents, manage children’s daily needs, provide emotional support, and respond to sudden illnesses and other conditions. Since children are in a critical stage of growth and development, and their tumors are characterized by high malignancy, disease complexity, early onset, prolonged chemotherapy cycles, and numerous adverse reactions, family caregivers must provide extensive care. Their preparedness and capacity play pivotal roles in reducing caregiving burdens and enhancing quality of life [4-6].

The family-centered empowerment model (FCEM), developed by Alhani et al [6] in 2003, aims to empower patients and their families by defining essential elements for improving chronic care outcomes [6]. This model builds on the family-centered care philosophy, which emphasizes the child’s family as the “center of capacity building” [6].

The FCEM involves a 4-step process. The first step consists of improving knowledge through educational programs using resources, such as posters, models, and handouts, as well as interactive methods, including quizzes and question-and-answer sessions. The second step is self-efficacy enhancement, the third step is the promotion of self-esteem through participation in educational activities, and the fourth step involves evaluating the entire intervention period [6]. By following this structure, FCEM helps caregivers acquire the necessary skills and confidence to manage their child’s condition effectively and maintain a balanced family life [6].

This model has also been widely used for patients with chronic diseases in China. Nonpharmacologic interventions based on the FCEM have demonstrated positive effects on the quality of life, knowledge, and self-efficacy of patients with chronic diseases, such as chronic obstructive pulmonary disease, thalassemia major, asthma, and myocardial infarction. These interventions also alleviate stress, anxiety, and restlessness in family caregivers [7,8]. While FCEM was initially developed for chronic illnesses, its structured framework is equally applicable to the caregiving needs of family caregivers of patients with malignant tumors [9].

Although studies highlight the positive impact of FCEM-based interventions on caregivers of patients with malignant neoplasms [7-9], most have focused on primary caregivers of adult patients. Compared with adults, children require more detailed and comprehensive care because of their younger age, longer chemotherapy cycles, and critical developmental stages. This necessitates caregivers with greater preparedness and advanced caregiving skills. The impact of the FCEM on caregiving capacity and preparedness among family caregivers of children with malignant neoplasms remains unclear.

### Objectives

This study aims to recruit family caregivers of children with malignant neoplasms to develop a corresponding intervention program based on the FCEM and to evaluate its impact on caregiving preparedness and caregiving capacity. Additionally, the study aims to examine the effects of the empowerment intervention on caregivers’ self-efficacy as well as their levels of depression, anxiety, and stress.

### Research Hypotheses

This study has the following research hypotheses:

1. Statistically significant differences in caregiver preparedness and caregiving capacity will occur after the FCEM intervention program.
2. Significant differences in self-efficacy, depression, anxiety, and stress will occur in the intervention group after the intervention compared to previous measurements.

## Methods

### Data Collection

#### Setting and Participants

This is a quasi-experimental study. This protocol is version 1.0, finalized on July 8, 2025. We will select children with malignant tumors and their family caregivers who were hospitalized at West China Second Hospital, Sichuan University, between July 2025 and December 2026. Family caregivers of the children will be enrolled. The researchers will provide a detailed description of the study’s purpose and content to the participants, who must sign an informed consent form.

**Figure 1:**
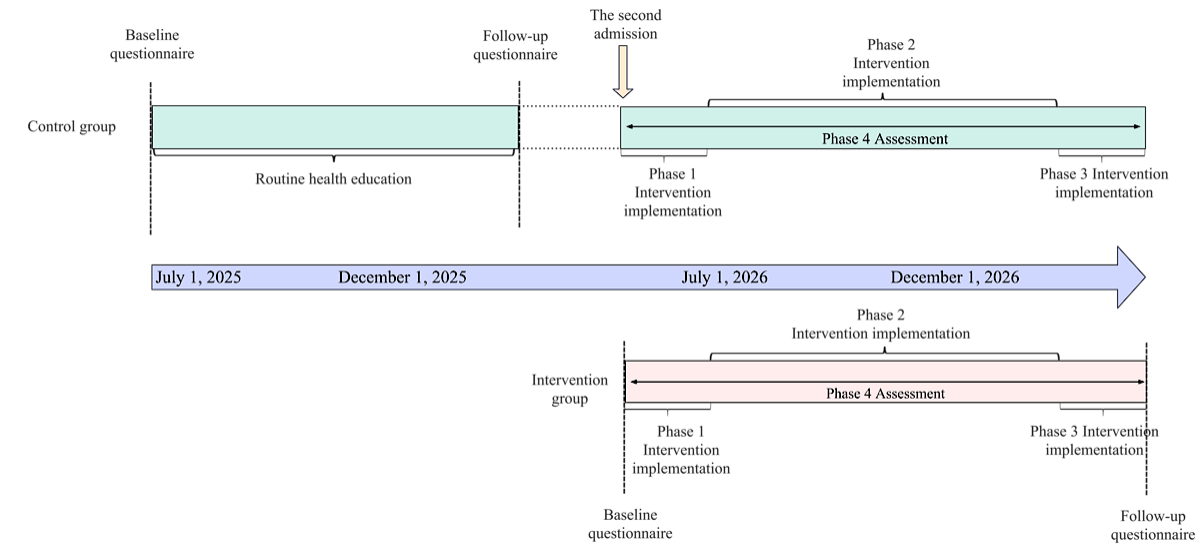
Participant timeline.

The QR code for the web-based questionnaire will be distributed after caregivers provide informed consent. The family caregivers recruited for the study will be divided into control and intervention groups. Children will be grouped according to their diagnosis and treatment timeline as follows: children admitted from July to December 2025 will form the control group, and those admitted from July to December 2026 will form the intervention group. The caregivers of the children will be enrolled along with the children. In the intervention group, the intervention program will be implemented after inclusion. For the control group, where routine health promotion is implemented, an intervention program based on FCEM will be offered upon the second admission to the hospital (Figure 1).

After the family caregivers complete the baseline survey, the researchers will distribute paperwork or provide verbal health promotion with relevant health promotion components and assess the caregivers’ knowledge weekly.

The questionnaire will be re-administered 3 days before the child is discharged. One researcher will enter the data, which will be double-checked for errors by another researcher. After all data are entered, the analysis will be performed by a designated specialist who remains unaware of the groupings. Finally, 2 additional researchers will independently review the analyses to ensure accuracy. Family caregivers will be aware of their grouping. If family caregivers prefer to adjust their groupings, appropriate adjustments will be made based on their identified needs.

The inclusion criteria are as follows: (1) children aged 0-18 years, (2) children clinically diagnosed with malignant neoplasms, and (3) first-time visitors to our hospital.

The exclusion criteria are as follows: (1) children diagnosed and treated with radiotherapy or chemotherapy in other hospitals and (2) children undergoing their first treatment in our hospital for recurrence of the disease.

Primary caregiver inclusion criteria are as follows: (1) the caregiver is enrolled along with the child, (2) the caregiver is aged 18 years or older, (3) the caregiver is a member of the child’s immediate family, (4) the caregiver has cared for the child for the longest period among multiple caregivers, (5) the caregiver has basic communication and reading skills and is proficient in Chinese, and (6) the caregiver has no previous history of psychiatric illness or consciousness-related disorders.

The sample size was calculated to be 128 using G*POWER 3 software [10] with the parameters α=.05, 1 – β=0.8, and effect size=0.5. Considering a 10% lost-to-follow-up rate, the total sample size required was estimated to be 142, with 71 caregivers in the intervention group and 71 in the control group.

#### Sociodemographic Variables

Sociodemographic variables in patients (including age, sex, ethnicity, medical payment method, clinical diagnosis, and duration of disease) and their caregivers (including age, relationship with the patient, ethnicity, place of residence, number of children, marital status, level of education, occupation, monthly family income, caregiving experience, number of other persons in need of care, hours of care per day, health status, disease awareness, and access to information) will be recorded. Disease-related variables will be obtained from the consent forms signed by the patients and their guardians.

#### Dependent Variables

The outcome variables are caregiving preparedness, caregiving capacity, self-efficacy, depression, anxiety, and stress. The tools used to measure these variables are described in Table 1.

The caregiver preparedness scale was developed by Archbold et al [11] to assess parents’ caregiving preparedness. This scale consists of 8 items, namely, preparedness for physical needs, emotional needs, service planning, caregiving stress, comfort care, coping with and managing emergencies, accessing medical information resources and help, and overall caregiving preparedness [11]. A 5-point Likert scale was used, with scores ranging from 0-4 indicating “not at all prepared” to “fully prepared,” and higher scores reflecting better preparedness for caregiving. Yanjin et al [12] adapted the scale for use in China, demonstrating its good reliability and validity as a suitable tool for assessing preparedness. The Cronbach α of the Chinese version was 0.92 [12].

**Table 1:** Measurement tools used to assess outcome variables.

Tool	Dimensions	Items
Caregiver preparedness scale	Physical care, emotional support, in-home support services, and caregiving stress	8
Family caregiver task inventory	Adaptation to caregiving roles, coping and assistance provision, management of personal emotions, caregiving needs, and assessment of family and social resources	25
General self-efficacy scale	Self-efficacy	10
Depression anxiety stress scale-21	Depression, anxiety, and stress	21

Caregiving capacity was measured using the family caregiver task inventory developed by Clark and Rakowski [13] in 1983, which covers 5 dimensions: role adaptation, caregiving assistance, emotional management, life adjustment, and use of social and family resources. It consists of a total of 25 items. The family caregiver task inventory uses a 3-point Likert scale, with 0 indicating “no difficulty,” 1 indicating “some difficulty,” and 2 indicating “great difficulty.” Higher scores represent greater difficulty and poorer caregiving capacity. The Cronbach α of the Chinese version was 0.86 [14].

The general self-efficacy scale was developed by Schwarzer et al [15] in 1981 and subsequently translated into Chinese [15,16]. The general self-efficacy scale assesses the self-efficacy of family caregivers. It contains 10 items within the single dimension of self-efficacy and is scored on a 4-point Likert scale, ranging from 1 (“not at all true”) to 4 (“exactly true”), with a total score of 10 to 40. Higher scores indicate better general self-efficacy [15,16]. The Cronbach α coefficient for this scale was 0.91 [15].

The depression anxiety stress scale-21 was developed by Lovibond and Lovibond [17] in 1995 and later translated into Chinese by Gong et al [18]. The depression anxiety stress scale-21 evaluates depression, anxiety, and stress levels in family caregivers. It contains 21 items across 3 dimensions: depression, anxiety, and stress. The depression subscale includes items 3, 5, 10, 13, 16, 17, and 21; the anxiety subscale includes items 2, 4, 7, 9, 15, 19, and 20; and the stress subscale includes items 1, 6, 8, 11, 12, 14, and 18. A 4-point Likert scale is used, with scores ranging from 0 (“did not apply at all”) to 3 (“applied most or all of the time”). Higher scores indicate higher levels of depression, anxiety, and stress. The Cronbach α coefficient of the scale was 0.89 [18].

#### Intervention Program

##### Intervention Content Development

Through a preliminary search of the literature, the caregiving needs of family caregivers of children with malignant neoplasms were identified to include disease information needs, such as knowledge about the disease, its progression, and prognosis; symptom management needs, including knowledge related to Peripherally Inserted Central Catheter (PICC) or infusion port care; dietary management needs; prevention and treatment of infections; management of daily living activities; prevention and treatment of adverse reactions to chemotherapy drugs; and psychological regulation needs, such as managing anxiety, nervousness, and fear.

We plan to select approximately 17 caregivers of children with malignant neoplasms for interviews using purposive sampling as part of the preliminary research. Face-to-face interviews using open-ended questions will be conducted by experienced interviewers. The interview is expected to last 15-30 minutes. It will be discontinued immediately if the child requires medical attention. The interview outline includes the following questions:

1. What kinds of feelings do you experience during caregiving, and what are your biggest concerns and stresses?
2. What problems or needs have you encountered in the caregiving process, and how have you coped with them?
3. Regarding treatment and care, what kind of health guidance would you like medical workers to provide (including knowledge about diseases, dietary management, daily life activities management, maintenance of PICCs and infusion ports, and knowledge of drug reactions)?

Data collection will be discontinued when thematic saturation is reached. Saturation is defined as the point at which no new themes or codes emerge from at least 2 consecutive interviews. The results of interviews with 17 caregivers will be summarized and refined to determine the main needs of the caregivers. The results of a previous pilot study on parents of children with acute lymphoblastic leukemia showed that the main needs of caregivers were in the following areas:

1. Disease information, including symptoms and manifestations, incidence rate, relapse rate, cure rate, 3-year to 5-year survival rate, and other disease-related details.
2. Infusion port and PICC maintenance, including precautions after infusion port implantation, PICC-related knowledge, and daily observation priorities.
3. Infection prevention, including methods for infection prevention and management.
4. Medication guidance, including common adverse drug effects and steps to observe and manage them.
5. Dietary guidance, including methods for preparing hygienic and appropriate meals for children.
6. Psychological aspects, including methods to alleviate negative emotions, such as anxiety and depression, in caregivers and children.

A draft intervention program will be developed based on the FCEM model, literature review, results of the preliminary research, and the departmental health education manual (Multimedia Appendix 1).

##### Intervention Revision

After the first draft is finalized, a group meeting is planned for the researchers and team members, all of whom are clinical and nursing experts in pediatric hematology. The group includes a hematologist and 5 nursing staff members with more than 5 years of experience. Before the study begins, a group meeting will be held to jointly negotiate the terminology to be used with patients during implementation and to ensure that the terminology used during implementation is harmonized.

#### Intervention Delivery Process

##### Intervention Group

The intervention group will be provided with a family empowerment intervention program based on usual care.

Three sessions of approximately 15-30 minutes each will be conducted within 3 days of the child’s admission to the hospital, from the fourth day to 3 days before discharge. The timing of the intervention sessions was determined based on evidence from a prior study conducted in China involving caregivers of children with asthma combined with clinical experience [19]. The researcher or charge nurse will assess the caregivers’ knowledge every week using uniform evaluation criteria and adjust the topic or number of interventions within each phase based on the knowledge gained, with the final intervention content and timeline remaining unchanged.

During stage 1 (within 3 days of admission), the researchers will explain the use of the facilities in the ward, including the proper use of a hospital bed or laminar flow bed, and outline safety precautions in the ward (including preventing falls, falls out of bed, and avoiding burns). They will also provide information about daily care, infection prevention, dietary considerations (eg, low-fat diets and special diets for children experiencing vomiting), input and output monitoring, the recognition of symptoms for oral and perianal infections and how to gargle and cleanse the perianal area, and disease-related information, including cure rates and recurrence rates. Completing stage 1 will require 15-25 minutes.

During stage 2 (fourth day after admission to 3 days before discharge) the nurse in charge will ask the main caregiver about difficulties and problems encountered during the caregiving process using open-ended questions, such as “In your opinion, what is the most difficult thing right now?” “Do you have difficulties taking care of your children and yourself?” “What kind of information would you like the nurse to provide more of while providing care for your child?” Based on the preinterview, the nurse will conduct adequate communication with the main caregiver in response to their difficulties, listen patiently, and provide positive guidance tailored to their psychological state. A childcare program will be developed, and the following content will be explained to the patient’s family, part of which will be presented in written or video form: (1) medication guidance based on the actual use of drugs by the child, including the dosage, frequency, and adverse effects of the drugs; (2) PICC-related knowledge, including daily observation, handling of abnormalities, prevention and identification of complications, and precautions for daily life and exercise; (3) postoperative care for infusion port implantation, including postoperative diet, postoperative medication changes, observation during use, and postdischarge precautions; (4) blood indicators, including an explanation of blood indicators, such as platelets, hemoglobin, and neutrophils, along with precautions to be taken in case of critical values; (5) condition monitoring and emergency treatment, including simple treatments in case of fever or other emergencies; and (6) psychological support, including sharing stories of other successful cases to positively motivate the caregivers. Completing stage 2 will require 20-30 minutes.

Stage 3 (3 days before discharge) will involve the evaluation of outcomes and discharge instructions as well as communication with the patient to address problems related to caregiving abilities and caregiving preparedness, affirm the level of care provided by the family caregiver during the child’s hospitalization, boost their confidence, and guide the child’s postdischarge medication, nursing care, review, and follow-up chemotherapy plans. Completing stage 3 will require 10-20 minutes.

Stage 4 encompasses the entire implementation process. During program implementation, a dedicated researcher will coach the primary caregiver 1-on-1 to ensure they understand the program content (ie, until they can repeat it independently). Family caregivers will be instructed to record changes in their children’s symptoms. The family caregivers’ knowledge of the program will be assessed weekly (Multimedia Appendix 2).

##### Control Group

In the control group, routine nursing care and current health education content will be provided, including the following: introduction to the department’s environment, rules, and regulations; admission counseling; examination guidance; ongoing education on daily care, medication management, and health practices during hospitalization; proactive support and communication by medical staff, including resolving questions and sharing caregiver experiences; and discharge instructions with follow-up schedules for chemotherapy (Multimedia Appendix 3).

Participants in the control group will be offered an FCEM-based intervention regimen on their second admission (at the beginning of the second course of treatment), which will be appropriately adapted to the control group’s second chemotherapy treatment.

##### Follow-Up Assessment

To evaluate the sustained impact of the intervention, an additional follow-up will be conducted 1 month after discharge. This will be completed via telephone and web-based questionnaire by the research assistants. The same validated instruments used during hospitalization will be used to measure caregiving preparedness, caregiving capacity, self-efficacy, and depression, anxiety, and stress.

### Data Analysis

Data will be analyzed using SPSS version 26.0 (IBM Corporation). For general information, measurement data conforming to a normal distribution will be described by means and SDs, and count data will be described by frequencies and constitutive ratios. Missing data will be removed and will not be included in the analysis. Participants who request to switch groups based on their preferences will be excluded from the primary analysis; however, we will summarize the baseline characteristics of these participants and compare them to those who remain in their originally assigned groups. This comparison will include demographics to assess the potential impact of nonrandom attrition on study validity.

Independent-sample *t* tests will be used to compare caregiving preparedness, caregiving capacity, self-efficacy, and depression, anxiety, and stress scores between the 2 groups before the intervention. After the intervention, both within-group and between-group differences in these variables will be analyzed using independent-sample *t* tests, paired *t* tests, Mann-Whitney tests, and paired rank-sum tests.

### Ethical Considerations

This study was approved by the Ethical Review Committee of West China Second Hospital of Sichuan University (approval number YXKY2024432). The trial was registered on February 5, 2025, with ClinicalTrials.gov (NCT06810388). Before data collection, the study leader will obtain written informed consent from all participants. Participants will also be informed in advance that they will be contacted for a follow-up assessment approximately 1 month after discharge.

During the study, personal information, such as names, gender, and other identifiers of participants and their children, will be replaced with codes or numbers to maintain confidentiality. Access to this information will be limited to authorized researchers, ensuring the privacy of participants and their children is fully protected. While the study results may be published in academic journals, no personal details of the participants will be revealed.

All participants will be informed of their right to withdraw from the study at any time and that completing the questionnaire will not affect their children’s medical or nursing care. All family caregivers in the control group will be asked if they would like to receive the family empowerment intervention program upon their second admission, which the researchers will provide.

## Results

### Recruitment Progress

The study was approved by the Ethical Review Committee of West China Second Hospital of Sichuan University on January 9, 2025 (approval number YXKY2024432). We plan to recruit participants for the control group from July to December 2025. We will recruit caregivers for the intervention group from July to December 2026. Data collection will occur throughout the recruitment period. Results are expected to be published in late 2027.

### Caregiving Preparedness and Capacity

We expect caregivers in the intervention group to have higher preparedness and caregiving capacity than those in the control group. In addition, significant improvement is expected within the intervention group after the intervention.

### Self-Efficacy Outcomes

Regarding the self-efficacy outcomes, caregivers receiving the intervention are likely to show increased self-efficacy after discharge. No major change is expected in the control group.

### Psychological Outcomes

Caregivers in the intervention group are expected to report significantly lower depression, anxiety, and stress scores after the intervention.

## Discussion

### Principal Findings

The intervention based on the FCEM model is expected to improve caregiving preparedness, caregiving capacity, and self-efficacy in family caregivers of children with malignant neoplasms. It may also reduce their psychological distress, including symptoms of depression, anxiety, and stress. These anticipated findings will contribute to enhancing the design and implementation of family-centered interventions in pediatric oncology care.

The results of this study will be shared with the Department of Pediatric Hematology and used to refine and improve existing health promotion strategies.

### Limitations

This study has several limitations. First, the intervention will be implemented for family caregivers of children with malignant neoplasms rather than targeting a specific cancer type, and caregivers of children with different cancer types may face distinct challenges. Second, the intervention period is relatively short, limiting the ability to observe the long-term effects of the FCEM intervention program on family caregivers. Finally, as this is a single-center study conducted at 1 hospital, the findings will reflect only the situation at this center. Considering geographic variations in the level of medical care, the findings may not be generalizable to primary care settings or populations with different cultural or socioeconomic backgrounds. In the future, the study could be expanded to include multiple centers. To further enhance the evaluation and adaptation of empowerment strategies, it is also recommended that future studies involve caregivers of children with various types of cancer.

### Strengths

The methodological strength of this study lies in the establishment of an intervention program tailored to the needs of family caregivers of children with malignant neoplasms. The program includes caregiving knowledge, skills, and psychological coping strategies to enhance the educational content of the family empowerment program. Designed for caregivers of children with various types of malignant neoplasms rather than a single type, the intervention program aims to contribute to a more comprehensive health education system and facilitate future replication.

### Future Directions

To further expand the impact of such interventions, future studies may consider integrating emerging technologies into caregiver support strategies. As digital health tools continue to evolve, there is growing interest in leveraging emerging technologies to enhance caregiver support [20,21]. With the rapid development of large language models, future studies may consider exploring the integration of large language model–based tools to further support family caregivers.

## Appendix

Multimedia Appendix 1 Theoretical framework mapping the family-centered empowerment model to the phases of the intervention.

Multimedia Appendix 2 Family caregiver mastery evaluation form (weekly).

Multimedia Appendix 3 Routine health education content for the control group.

Multimedia Appendix 4 Sample informed consent form.

## Abbreviations

FCEM: family-centered empowerment model
PICC: peripherally inserted central catheter
