# Supplementary figures and images for: Effect of a Family-Centered Empowerment Model–Based Intervention on the Caregiving Capacity and Preparedness of Caregivers of Children With Malignant Neoplasms: Protocol for a Quasi-Experimental Study
Source: JMIR Res Protoc. 2025 Jul 29;14:e73304. doi: 10.2196/73304 (PMC12344386; doi:10.2196/73304)

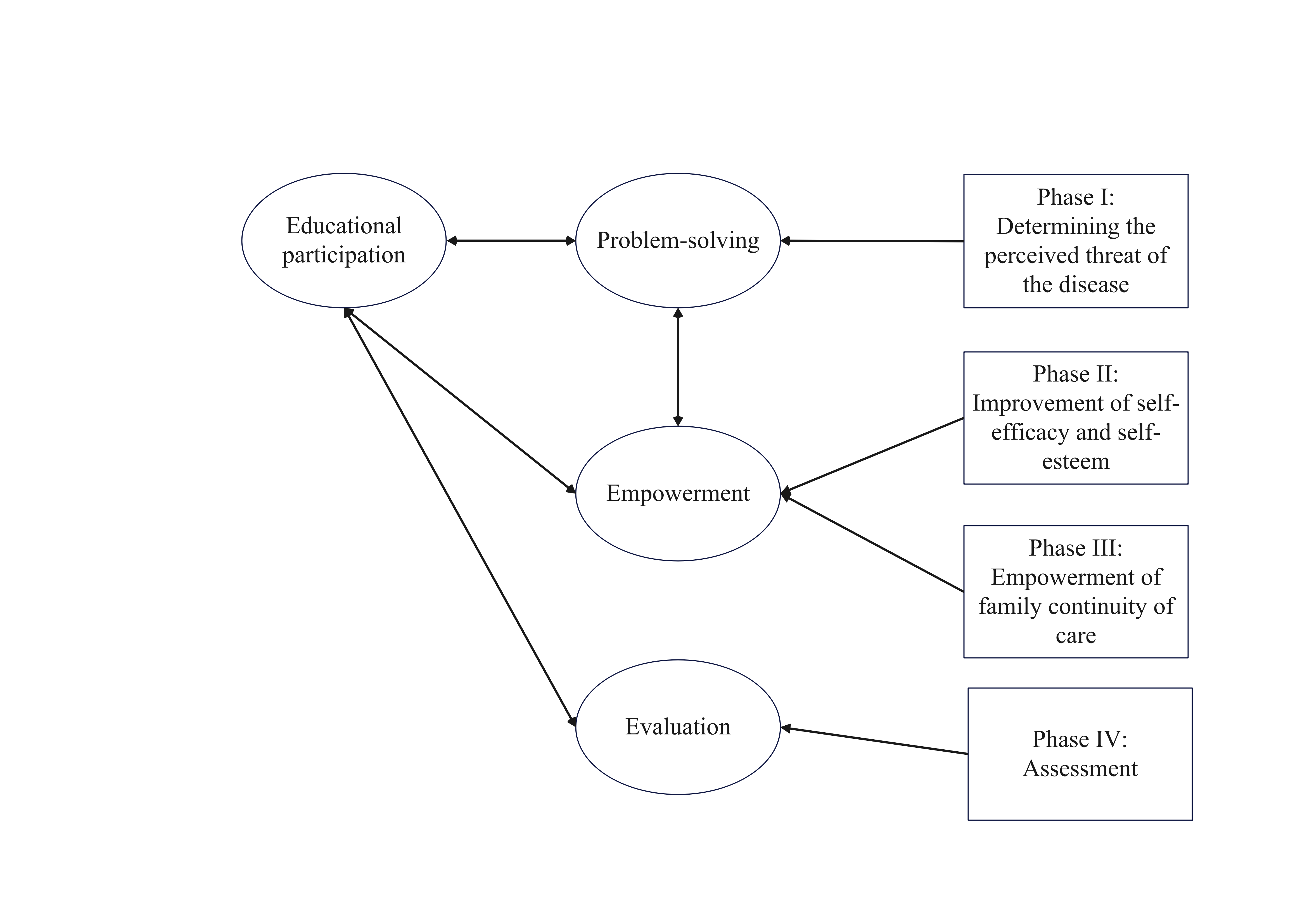

Supplement: Multimedia Appendix 1 [file resprot_v14i1e73304_app1.png]
