# Supplementary material for: Effect of a Family-Centered Empowerment Model–Based Intervention on the Caregiving Capacity and Preparedness of Caregivers of Children With Malignant Neoplasms: Protocol for a Quasi-Experimental Study
Source: JMIR Res Protoc. 2025 Jul 29;14:e73304. doi: 10.2196/73304 (PMC12344386; doi:10.2196/73304)
Supplement: Multimedia Appendix 2 [file resprot_v14i1e73304_app2.docx]

**Appendix II Family Caregiver Mastery Evaluation Form (Weekly)**

**Name of the child:__________ Name of caregiver:___________ Date of evaluation:___________ Evaluator:__________**

| **Items** | **Questions** | **Not mastered** | **Mastered** |
| --- | --- | --- | --- |
| 1. Use of hospital beds (or laminar flow beds) | Can you tell me how laminar flow beds work? |  |  |
| 1. Disease information | Can you tell me how much you learned about the disease? |  |  |
| 1. Dietary care | Can you tell me about kids' dietary taboos? What are the foods in a low-fat diet? What should you do if your child is vomiting? |  |  |
| 1. Input/output monitoring | Can you tell me how you count urine or in and out? |  |  |
| **Items** | **Questions** | **Not mastered** | **Mastered** |
| 1. Oral daily observation and care, perianal daily observation and sitz bath care | Can you tell me how you perform daily oral observations, oral cleansing, perianal status observations, and sitz baths on your children? |  |  |
| 1. Medication | Can you tell me what medicines the children will be taking today (checking against the doctor's orders) and how many times a day? Do you know any possible side effects of taking this type of medicine? |  |  |
| 1. PICC-related information | Could you tell me how you observe the puncture site daily and how you measure the child's arm or leg circumference? How should you carry out daily activities after a PICC puncture? |  |  |
| **Items** | **Questions** | **Not mastered** | **Mastered** |
| 1. Postoperative precautions for infusion port implantation | Can you tell me how you look around the puncture site of the infusion port needle daily? How often is the infusion port needle changed? How often is the dressing on the port changed? How long does it take to remove the stitches from a newly placed infusion port? How often do you need to maintain your child’s infusion port? |  |  |
| 1. Understanding of blood indicators | Can you tell me what haemoglobin, platelets, and neutrophils represent? What do you look for when these values are abnormal? |  |  |
| (10) Disease surveillance and emergency response | Can you tell me what you would do if the child has a fever or vomits? |  |  |

**Assessment rules:** The caregiver is asked questions about each item using a "one question, one answer" format. For example, “Do you know how to use a laminar flow bed?” If the caregiver answers accurately, it will be regarded as "mastered," and the nurse in charge of the assessment will mark "✓" in the Mastered column. If the caregiver fails to answer accurately or is unable to answer, it will be regarded as "not mastered." Detailed notes must be made in the Not Mastered column, including specific areas not fully understood. Reassessment is conducted 3–7 days after a previous failure.
