# Supplementary material for: Effect of a Family-Centered Empowerment Model–Based Intervention on the Caregiving Capacity and Preparedness of Caregivers of Children With Malignant Neoplasms: Protocol for a Quasi-Experimental Study
Source: JMIR Res Protoc. 2025 Jul 29;14:e73304. doi: 10.2196/73304 (PMC12344386; doi:10.2196/73304)
Supplement: Multimedia Appendix 3 [file resprot_v14i1e73304_app3.docx]

**Appendix III Routine Health Education Content for the Control Group**

| **Content Module** | **Main Content** | **Form of Implementation** | **Period** |
| --- | --- | --- | --- |
| **Introduction to Environment and Regulations** | Introduction to the ward environment, room layout, and hospital rules and regulations | Verbal explanation by nurse | Upon admission |
| **Admission Education and Examination Guidance** | Guidance on admission procedures and required medical examinations | Verbal explanation by nurse | On the day of admission |
| **Daily Care Education** | Ongoing education by nurses, including:  – Daily care techniques  – Medication use and precautions  – Hygiene and dietary guidance  – Sleep and emotional support | Bedside education by nurse | Throughout hospitalization |
| **Communication** | Doctors and nurses actively respond to caregivers’ questions | Informal communication | If necessary |
| **Sharing of Caregiving Experiences** | Informal sharing of caregiving experiences among family members in the ward or lounge areas | Spontaneous interactions | If necessary |
| **Discharge Guidance** | Nurses provide the following guidance before discharge:  – Post-discharge medication plan  – Home care precautions  – Chemotherapy and follow-up schedule | Bedside education by nurse | One day before discharge |
