# Supplementary material for: Effect of a Family-Centered Empowerment Model–Based Intervention on the Caregiving Capacity and Preparedness of Caregivers of Children With Malignant Neoplasms: Protocol for a Quasi-Experimental Study
Source: JMIR Res Protoc. 2025 Jul 29;14:e73304. doi: 10.2196/73304 (PMC12344386; doi:10.2196/73304)
Supplement: Multimedia Appendix 4 [file resprot_v14i1e73304_app4.doc]

**《基于以家庭为中心的赋权模式的干预方案对于恶性肿瘤患儿的家庭照顾者的照顾能力及护理准备度的影响》**

**研究参与者知情同意书•告知页**

**（**版本号：V1.0，版本日期：2024.11.14**）**

**亲爱的研究参与者：**

我们将要开展一项 《基于以家庭为中心的赋权模式的干预方案对于恶性肿瘤患儿的家庭照顾者的照顾能力及护理准备度的影响》 课题研究，您孩子的情况可能符合该项研究的入组条件，我们将邀请您及您的孩子参加该课题研究。本研究已经通过四川大学华西第二医院医学科研伦理委员会审核，同意进行研究。

在您和您孩子决定是否参加这项研究之前，请尽可能仔细阅读以下内容。它可以帮助您和您的孩子了解该项研究以及为何要进行这项研究，研究的程序和期限，参加研究后可能给您及孩子带来的益处、风险和不适。如果您愿意，您也可以和您的亲属、朋友一起讨论，或者请研究者给予解释，帮助您做出决定。

1. **研究任务来源：**本研究为自拟课题，暂未获得任何外部资助，由研究团队自主筹措资金进行。
2. **研究目的：**本研究以恶性肿瘤患儿的家庭照顾者为研究对象，基于以家庭为中心的赋权模式制定相应的干预方案，以阐明基于家庭为中心的赋权模式的干预方案对家庭主要照顾者的护理准备度及照顾能力的影响。
3. **研究时限： 2025年 7月 1日至 2026年 12月 1日**
4. **研究方案简介**

**4.1研究背景**

儿童期恶性肿瘤通常指发病年龄在0-14岁之间的恶性肿瘤，是造成儿童疾病死亡的第一大原因。儿童肿瘤疾病具有恶性程度高、病情复杂的特点，由于发病年龄较小、化疗周期长、不良反应较多，且患儿自我照护能力有限，对于家庭照顾者的依赖性较大。以家庭为中心的赋权模式（Family-Centered Empowerment Model, FCEM）是由Alhani等人开发的慢性病赋权模式。该模式是 “以家庭为中心的护理理念”的延续和发展，强调以儿童家庭为“能力建设中心”。以家庭为中心的赋权为家庭照顾者提供了获取知识和技能的机会，使他们能更好的管理家庭生活，从而提高所有家庭成员的生活方式和生活质量。

**4.2主要内容**

（1）阐明基于以家庭为中心的赋权模式的干预方案对恶性肿瘤患儿家庭照顾者的照顾能力及护理准备度的影响。

（2）探讨基于以家庭为中心的赋权模式的干预方案对恶性肿瘤患儿家庭照顾者的自我效能感、抑郁、焦虑、压力的影响。

**4.3 生命科学与医学研究伦理原则与要求**

本临床研究将遵循世界医学大会《赫尔辛基宣言》和《涉及人的生命科学与医学研究伦理审查办法》等相关规定。每位研究参与者在入选本研究之前，负责签署知情同意的研究者要以书面文字形式向其完整、全面地介绍本研究的目的、性质、程序以及可能的受益及风险等，并让研究参与者知晓他们有权随时退出研究。入选前，每位研究参与者均被充分知情，且有充分的时间考虑是否参加。研究参与者自愿参加并签署知情同意书之后方可纳入本研究。入选后，研究参与者随时可以拒绝或退出本研究。

1. **研究计划招募的研究参与者人数及纳入/排除标准：**

本研究只在四川大学华西第二医院单中心开展，共计划招募142名家庭照顾者作为研究参与者。

5.1患儿纳入标准：①患儿年龄0-18岁，②经临床诊断为恶性肿瘤。

主要照顾者纳入标准：照顾者虽患儿入组，①年龄≥18岁；②患儿的直系亲属；③多名照顾者选取照顾时间最长者；④具有基本的沟通和阅读能力，能够熟练使用中文；⑤既往或目前无精神病史或意识相关障碍。

5.2研究分组设置与分组方式：使用G POWER软件计算出样本量为128人α=0.05， 1-β= 0.8， effect size= 0.5）；考虑10%的失访率，总样本量为142人,其中干预组71人、对照组71人。

1. **研究期间，您需要做的是：**在本研究中，我们将会在各个阶段向您以书面或者口头形式提供各类健康教育，在您入院后三日内以及出院前一天分别发放调查问卷来进行调查研究，研究参与者需要填写按照实际情况来填写，大概需要10-15分钟。
2. **参加此项研究可能的风险和受益：**

7.1研究风险与防范：本研究属于类实验研究，采用的干预方案为非药物性干预方案，将不涉及患者的诊疗方案。在调查过程中，我们会尽全力保护研究参与者提供的信息不被泄露。本研究中我们的干预方案或调查问卷所涉及的一些问题可能会让研究参与者感到不舒服，研究参与者任何时候都可以拒绝回答此类问题或拒绝填写问卷，在研究中任何时刻，参与者都可以退出本研究。

7.2研究受益：本研究为类实验研究，该非药物性干预方案将不影响研究参与者的诊疗方案，故无受益改变，但您的参与可能会给今后提高恶性肿瘤患儿的家庭照顾者的护理准备及护理能力带来益处，为我科今后构建更为完善的健康宣教体系提供有益的信息。

1. **费用与补偿：**本研究为类实验研究，所使用的干预方案为非药物性干预方案，研究参与者不需要支付与研究相关的任何费用。本研究将确保研究参与者在参与研究过程中的权益得到充分保障。
2. **其他备选治疗方案**：本研究为类实验研究，拟采用的干预方案为非药物性干预方案，因此本研究将不会干预您孩子的临床常规治疗，您及孩子也可以选择不参加本项研究，这对您孩子日常所获得的常规治疗及护理不会带来任何不良影响。
3. **自愿参与原则：**是否参加研究完全取决于您的自愿，您可以拒绝参加研究。您有权在研究的任何阶段随时退出研究而不会遭到歧视或报复，您的任何待遇与权益也不会受到影响。如果您决定退出本研究，请与研究者联系，以便妥善诊疗疾病。

出于对您最大利益的考虑，如果您需要其他治疗，或者您没有遵守研究计划，或者发生了与研究相关的风险超过可能的受益或者有其他可能影响研究进行原因，研究者可以在研究过程中中止/终止您继续参加本项研究。如果发生本研究提前终止的情况，我们将及时通知您，您的研究人员会根据您的健康状况或研究现状为您提供妥善建议。

1. **研究参与者信息及隐私的保护/保密措施：**此项研究资料将按照规定保存在四川大学华西第二医院，研究者、研究主管部门、伦理审查委员会可查阅研究资料。任何有关本项研究结果的公开报告将不会披露您的个人身份，除非获得您的许可。我们将在法律允许的范围内，尽一切努力保护您个人研究资料的隐私和个人信息。
2. **更多信息的获取：**在参加本课题研究前，您应该尽可能多了解研究的情况；在研究过程中，您可随时了解与本研究有关的信息资料和研究进展。如果您有任何与本项研究有关的疑问或不理解的事情，您随时可向课题的研究者提出。在研究过程中，如有任何重要的新信息，可能影响您继续参加研究的意愿时，研究者会及时通知您；有关伦理和您的权益事宜可联系四川大学华西第二医院医学科研伦理委员会，联系电话：028-88570104。

**《基于以家庭为中心的赋权模式的干预方案对于恶性肿瘤患儿的家庭照顾者的照顾能力及护理准备度的影响》**

**知情同意书•同意签字页**

研究参与者声明：

本研究发起者详细、通俗告知了上述事项，并回答了我的全部提问；我已充分理解我在本研究中的权利、受益、义务和风险。经充分考虑后，我及我的孩子自愿成为本研究的参与者，将我及我孩子过去和现在病史及诊治情况告知研究者，并积极与研究者配合，进行本研究。

研究参与者（患儿）姓名：

研究参与者（主要照顾者）签名： 日期:

研究者声明：

作为研究者，我已经向研究参与者（或其监护人）详细说明了本研究的情况，包括研究的目的、程序、风险与获益、参与原则及资料保密等情况，给予他/她足够的时间阅读知情同意书、与他人讨论，同时我已回答了其所提出的所有问题。我已告知研究参与者（或其监护人）可随时退出研究，而且不会影响以后的治疗。

研究者签名： 日期：

研究者联系方式：
